# Intercalary allograft reconstruction following femoral tumour resection: mid- and long-term results and benefits of adding a vascularised fibula autograft

**DOI:** 10.1186/s12957-022-02650-x

**Published:** 2022-06-13

**Authors:** Vincent Crenn, Yonis Quinette, Charlie Bouthors, Gilles Missenard, Brice Viard, Philippe Anract, Stéphane Boisgard, Eric Mascard, François Gouin, The Société d’Orthopédie de l’Ouest

**Affiliations:** 1 Service de Chirurgie Orthopédique, CHU de Nantes, Place Alexis Ricordeau, 44000 Nantes, France; 2 INSERM, UMR 1238, Phy-Os, Université de Nantes, Nantes, France; 3 Service de Chirurgie Orthopédique, APHP/CHU Kremlin-Bicêtre, 78 rue du Général Leclerc, 94270 Le Kremlin-Bicêtre, France; 4 Service de Chirurgie Orthopédique, AP-HP/Hôpital Cochin, 27 rue du Faubourg Saint-Jacques, 75014 Paris, France; 5 Service de Chirurgie Orthopédique, CHU Gabriel Montpied, 58 rue Montalembert, 63000 Clermont-Ferrand, France; 6 Département de Chirurgie Orthopédique Pédiatrique, APHP/Hôpital Necker, 149 rue de Sèvres, 75015 Paris, France; 7 Département de Chirurgie, Centre Léon Bérard, 28 rue Laennec, 69008 Lyon, France

**Keywords:** Intercalary allograft, Vascularised fibula graft, Bone tumour, Bone reconstruction

## Abstract

**Purpose:**

Bone healing in femoral reconstructions using intercalary allografts can be compromised in a tumour context. There is also a high revision rate for non-union, infection, and fractures in this context. The advantages and disadvantages of an associated vascularised fibula graft (VFG) are still a matter of debate.

**Methods:**

In a multicentre study, we retrospectively analysed 46 allograft reconstructions, operated on between 1984 and 2017, of which 18 were associated with a VFG (VFG+) and 28 without (VFG−), with a minimum follow-up of 2 years. We determined the cumulative probability of bone union as well as the mid- and long-term revision risks for both categories by Kaplan-Meier survival analysis and a multivariate Cox model. We also compared the MSTS scores.

**Results:**

Significant differences in favour of VFG+ reconstruction were observed in the survival analyses for the probability of bone union (log-rank, *p* = 0.017) and in mid- and long-term revisions (log-rank, *p* = 0.032). No significant difference was observed for the MSTS, with a mean MSTS of 27.6 in our overall cohort (*p* = 0.060). The multivariate Cox model confirmed that VFG+ was the main positive factor for bone union, and it identified irradiated allografts as a major risk factor for the occurrence of mid- and long-term revisions.

**Conclusion:**

Bone union was achieved earlier in both survival and Cox model analyses for the VFG+ group. It also reduced the mid- and long-term revision risk, except when an irradiated allograft was used. In case of a tumour, we thus recommend using VFG+ from a fresh-frozen allograft, as it appears to be a more reliable long-term option.

**Supplementary Information:**

The online version contains supplementary material available at 10.1186/s12957-022-02650-x.

## Introduction

The femur is a common site for primary bone tumours, but a diaphyseal location is less common than at the extremities [1]. Several techniques have been described for reconstruction after extensive diaphyseal femoral resection (> 10 cm) [2]: biological reconstructions: vascularised autograft [3], Masquelet’s induced membrane [4], allograft [5], combined graft [6], or even distraction osteogenesis or transport techniques [7], and finally prosthetic reconstructions using segmental prostheses, or custom reconstructions [8–11]. Some other innovative biologic techniques are also described but with a short follow-up on a small cohort [12]. Intercalary prostheses require a large residual bone segment to secure their stems and are, therefore, limited by the extent of the resection. Moreover, long-term survival is questionable with these implants. Although they allow early weight-bearing, the rate of complications is high (up to 60%), consisting primarily of infections, fractures, and loosening [11, 13]. Several authors also report the use of devitalised tumour-bearing bone with interesting results, but with tumour histological analysis potential issues [14–16]. Vascularised fibular autograft reconstructions alone are mainly used in children, with encouraging results, but the non-weight-bearing period, which is necessary for graft hypertrophy and consolidation, is long and significantly at risk of fracture (up to 15%) [17, 18]. This technique also has additional morbidity due to the second surgical site [19, 20]. In adults, allograft reconstructions, commonly used in extensive diaphyseal reconstructions, are favoured. Their mechanical qualities are similar to those of normal bone, with the possibility of using a graft of the same bone and with a similar calibre, albeit with a risk of non-union, fatigue fracture due to the lack of integration, or even resorption [21].

It is common for an autograft to be associated with the femoral allograft. This can be a vascularised fibula graft (VFG) [22–24] or other types of bone graft such as a rectangular cortical cancellous tibial graft (TG) [25] or a cancellous iliac crest graft (ICG). There are many ways to stabilise the allograft as well as ways to prepare and conserve the allograft (irradiated, fresh-frozen) [2, 26, 27]. The ideal approach has to be established in terms of bone union rates, revisions, and functional outcomes, especially after substantial follow-up. Ultimately, although the indications for intercalary allograft femoral reconstructions are increasingly standardised, the use of an associated graft is still debatable. This subject is particularly critical for the femur, which is a bone that is subject to pronounced compressional stress and rotational constraints. Since it concerns young patients with long life expectancies, it is essential that this specific topic is evaluated in mid- and long-term follow-up.

Our hypothesis is that adding a VFG may improve bone healing and the longevity results of allograft reconstructions in femoral resections. Therefore, we analysed whether adding a VFG (1) improved bone healing and (2) limited the rate of mid- and late-term revisions. We also sought to determine the functional impact of a VFG in this context. In addition, a multivariate analysis was carried out to identify whether other factors, such as the stabilisation modalities or the type of allograft, influence bone union and mid/late-term revisions.

## Materials and methods

### Patients

This retrospective study was conducted in four French hospitals specialised in oncological orthopaedic surgery, as part of the work resulting from the topic on allografts at the symposium of the French Society of Orthopaedic and Trauma Surgery. All consecutive whole-circumference resections of primary tumours in the long bones that were reconstructed with an intercalary allograft of at least 10 cm were included and reviewed retrospectively. Patients operated on between October 1984 and February 2015 with a minimum follow-up of 2 years were included. Patients with epiphysis or joint invasion were excluded.

### Methods

Allografts were harvested under sterile conditions during post-mortem tissue donation and were stored at − 80 °C by our dedicated bone banks. The allografts were fresh-frozen or irradiated. All of the patients received perioperative antibiotics according to the relevant protocols.

During the surgical tumour excision, the femoral allograft was kept in a saline solution containing antibiotics. The patients also received intraoperative intravenous antibiotic prophylaxis. The allografts were prepared using cryopreservation or irradiation and freezing. All of the resections spared the articular surface, with preservation of the epiphysis.

**Fig. 1:**
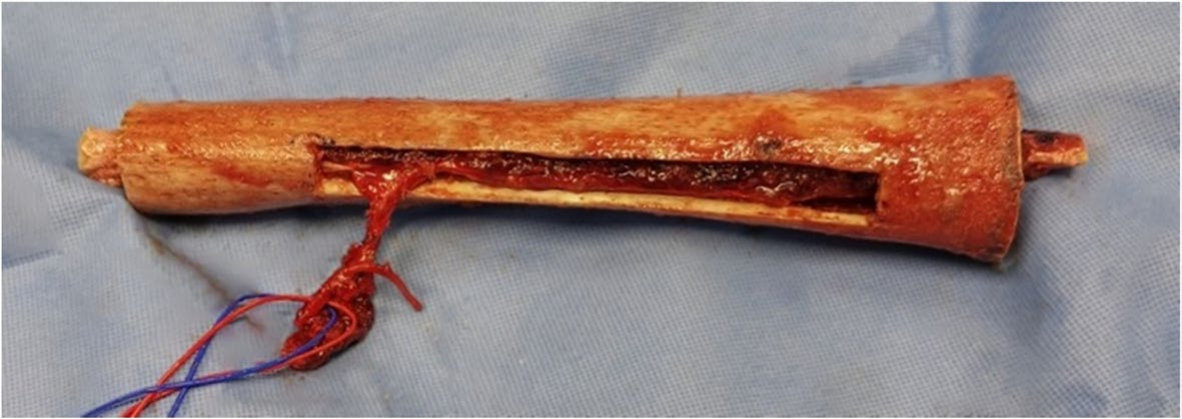
Vascularised fibula embedded in the femoral allograft before implantation. The allograft window allows vascular anastomosis according to the Capanna technique [15]

In the case of an associated VFG, the fibula was harvested in a standard manner by a second team using a lateral approach from the contralateral limb. The pedicle was then anastomosed termino-laterally on the artery and femoral vein. Depending on the case, the VFG could either be affixed with screws or embedded in the allograft (Fig. 1). In cases that did not involve an associated VFG, a tibial graft (TG) was harvested at the same time, on the homo- or contralateral side. A cancellous bone graft (CBG) from the iliac crest or reaming product was also an option associated with the allograft.

#### Evaluation methods

The aim of this study was to assess the impact of adding a VFG. Therefore, the main objective was to study the bone-healing time, and the primary criterion was defined by a cortical union of at least 75%, as analysed on standard X-rays. If the assessment of union was inconclusive on conventional X-rays, the union was assessed using computed tomography (CT). Surgical intervention to facilitate the union of osseous junctions in Henderson type 2 complications, at least 6 months after the primary surgery, was defined as non-union.

The secondary criteria comprised mid- and long-term revisions (occurring after 2 years of follow-up), defined as any surgical revision at the surgical site of the allograft reconstruction, excluding oncological resection. These complications were investigated according to the modified Henderson classification of ISOLS [28]. We also analysed the functional results, based on the MSTS scores [29], and we applied a multivariate Cox model to bone union and mid- and long-term revision to assess other variables.

#### Statistics and ethics

The statistical analyses were carried out using the IBM SPSS Statistics V19 software. The survival analyses were conducted using the Kaplan-Meier method, with comparisons based on the log-rank test [30]. Univariate and multivariate Cox proportional hazard regression analyses were used and performed with a stepwise forward conditional method. Comparative analysis of the data between the subgroups was conducted using the chi^2^ or Fisher’s exact tests and Student’s *t*-test. The threshold for significance was *p* < 0.05. The institutional review board approved the protocol. In keeping with French legislation regarding retrospective anonymised study data (articles L.1121-1 paragraph 1 and R1121-2, Public Health Code), Nantes University Hospital confirmed that approval from the ethics committee was not required given the non-interventional nature of the study, and no ethics committee approval was required at the time that the study was started. The database was anonymised, and all of the patients provided their verbal consent and received an information document.

## Results

**Fig. 2:**
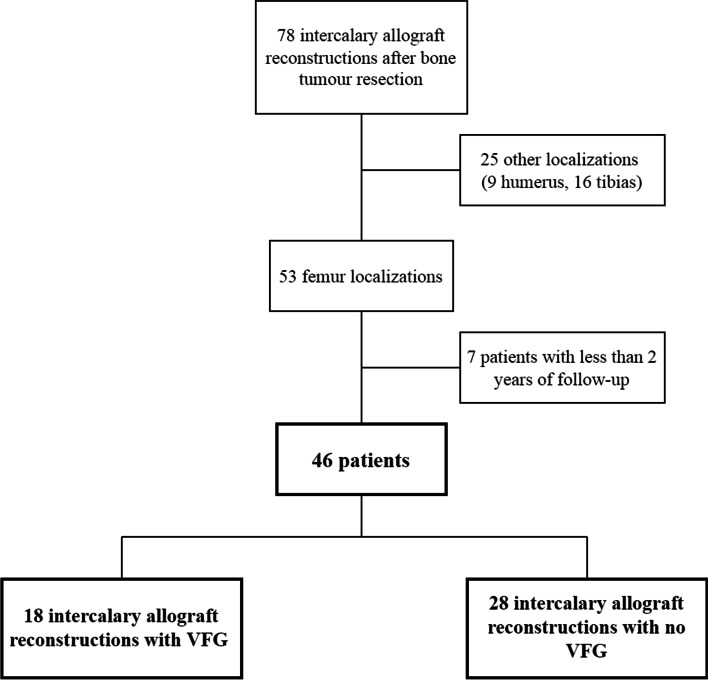
Study flowchart

Of the 78 patients operated on for an intercalary allograft reconstruction, 46 patients were included: 18 (39.1%) were operated on with VFG (VFG+) and 28 (60.9%) with no VFG (VFG-) (Fig. 2).

The average follow-up was 132.7 months, with the follow-up exceeding 10 years for 15 patients (32.6%) and 25 years for 3 patients (6.5%). No significant difference in the follow-up was noted between the VFG+ group and the VFG− group (114.9 months (37–233) vs. 145.8 months (24–402), *p* = 0.25) (Table 1). The VFG− group comprised two other graft modalities: 19 associated TG (67.9%) and 9 CBG (32.1%).

**Table 1:** Description of the population of the entire cohort and comparison of the qualitative data between the VFG+ and VFG− subgroups using the chi^2^ or Fisher’s exact test and for the quantitative data the *t*-test

	Cohort, ***n*** = 46	VFG+, ***n*** = 18 (39.1%)	VFG−, ***n*** = 28 (60.9%)	*p*-value
**Demographic data**				
Age (years)	20.9 (7–62)	19.6 (7–48)	21.7 (9–62)	0.553
Male	16 (34.8%)	8 (44.4%)	8 (28.6%)	0.270
Female	30 (65.2%)	10 (55.6%)	20 (71.4%)	
Follow-up (months)	132.7 (24–402)	114.9 (37–233)	145.8 (24–402)	0.250
Resection length (mm)	198 (100–340)	190 (140–260)	203 (100–300)	0.395
**Diagnosis**				0.088
Osteosarcoma	26 (56.5%)	9 (50.0%)	17 (60.7%)	
Ewing’s sarcoma	11 (23.9%)	7 (38.9%)	4 (14.3%)	
Chondrosarcoma	5 (10.9%)	0 (0.0%)	5 (17.9%)	
Others^a^	4 (8.7%)	2 (11.1%)	2 (7.1%)	
**Associated therapies**				
Adjuvant chemotherapy	35 (76.1%)	16 (88.9%)	19 (67.9%)	0.160
Adjuvant radiotherapy	4 (8.7%)	2 (11.1%)	2 (7.1%)	0.639
**Allograft preparation**				**0.022**
Irradiated	14 (30.4%)	2 (11.1%)	12 (42.9%)	
Fresh-frozen	32 (69.6%)	16 (88.9%)	16 (57.1%)	
**Stabilisation device**				**< 0.001**
Nail	12 (26.1%)	9 (50.0%)	3 (10.7%)	
Bridging plate	17 (37.0%)	9 (50.0%)	8 (28.6%)	
Nail + plate	17 (37.0%)	0 (0.0%)	17 (60.7%)	

^a^In the VFG group: one fibrosarcoma, one undifferentiated carcinoma; in the no VFG group: one clear cell renal carcinoma, one haemangioendothelioma

There were significantly more irradiated allografts in the VFG− group compared to the VFG+ group (12 (42.9%) vs. 2 (11.1%); *p* = 0.022). Lastly, the resorption scores according to the ISOLS classification were significantly worse in the VFG− group, with 25% of the patients being scored 3 or 4 compared to 0% in the VFG+ group (*p* = 0.032) (Table 2).

**Table 2:** Description of the population based on the postoperative follow-up data and comparison of the qualitative data between the VFG+ and VFG− subgroups using the chi^2^ or Fisher’s exact test and the quantitative data using the *t*-test

	Cohort, ***n*** = 46	VFG+, ***n*** = 18 (39.1%)	VFG−, ***n*** = 28 (60.9%)	*p*-value
**Revision surgery**				0.116
*n* = 0	19 (41.3%)	10 (55.6%)	9 (32.1%)	
*n* = 1; 2	15 (32.6%)	5 (27.8%)	10 (35.8%)	
*n* ≥ 3	12 (26.1%)	3 (16.6%)	9 (32.1%)	
**Allograft resorption (ISOLS)**				**0.032**
1, excellent; 2, good	39 (84.8%)	18 (100%)	21 (75.0%)	
3, fair; 4, poor	7 (15.2%)	0 (0.0%)	7 (25.0%)	
**Complications**				
Henderson 1	1 (2.2%)	1 (5.6%)	0 (0.0%)	0.391
Henderson 2	17 (37.0%)	6 (33.3%)	11 (39.3%)	0.683
Henderson 3	9 (19.6%)	2 (11.1%)	7 (25%)	0.448
Henderson 4	3 (6.5%)	1 (5.6%)	2 (7.1%)	1
Allograft removal^a^	2 (4.3%)	1 (5.6%)	1 (3.6%)	1
**Bone union**				
Healed at last follow-up	43 (93.5%)	17 (94.4%)	26 (92.9%)	1
Healed with no revision	28 (60.9%)	12 (66.7%)	16 (57.1%)	0.578
Mean bone union delay (months)	21.8 (4–120)	12.6 (5–34)	27.9 (5–120)	**0.012**
**Functional score**				
MSTS (/30) (*n* = 44)	27.3 (18–30)	26.3 (21–30)	28 (18–30)	0.060

^a^Excluding oncological causes

Two patients (4.3%) required allograft removal for non-oncological reasons. One (3.6%, VFG− group) underwent amputation due to septic complications 36 months postoperatively, and they died of oncological disease; another patient (5.6%, VFG+ group) required allograft removal and arthrodesis for an allograft fracture in non-union at 107 months of follow-up. We identified two specific complications of fibular harvesting: compartment syndrome requiring aponeurotomy at day 1 and an acquired toe claw that necessitated tendon lengthening surgery.

**Fig. 3:**
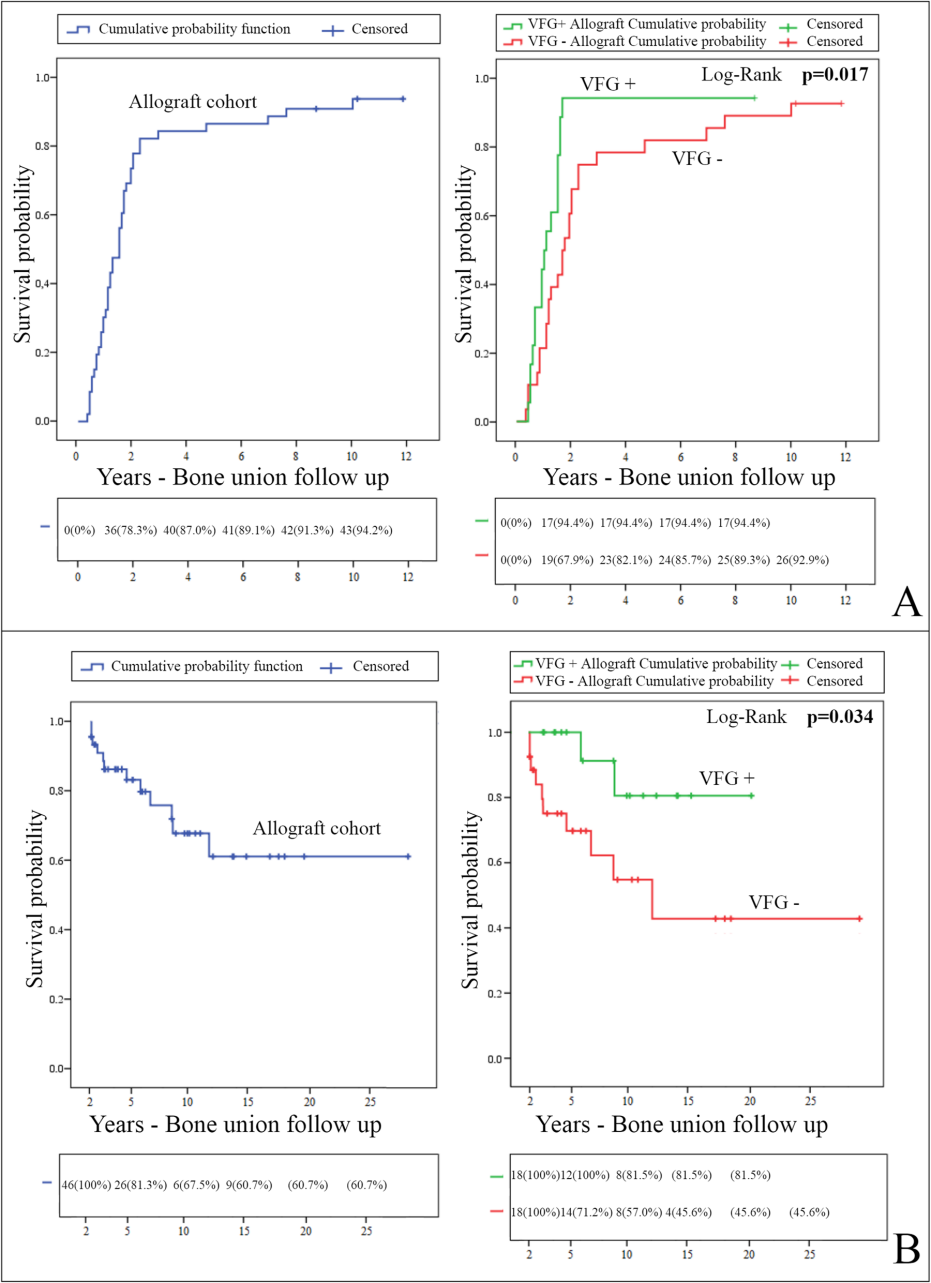
Kaplan-Meier cumulative probability curves. **A** Kaplan-Meier cumulative probability curves for allograft bone union in the overall cohort and log-rank comparison of the VFG+ and VFG− groups. Complete follow-up and follow-up excluding revisions in the first 2 years. The estimated median time for the bone union was 12.0 months (95% CI, 7.8 to 16.2) for the VFG+ group vs. 20.0 months (95% CI, 14.8 to 25.2) for the VFG− group. **B** Kaplan-Meier survival curves with failure defined as allograft revision for the VFG+ and VFG− groups, excluding carcinologic causes. Excluding revisions in the first 2 years: global and comparative follow-up

The overall bone union rate for the cohort at the last follow-up was 93.5%, with 43 patients healed out of 46, and the median bone union rate was 18.0 (95% CI, 13.6 to 22.4%) (Fig. 3A).

Using the log-rank test, a significant difference was observed between the VFG+ group and the VFG− group (*p* = 0.017). At 2 years, all but one of the patients achieved bone fusion (*n* = 17/18, 94.4%, 95% CI, 62.7 to 99.2%) in the VFG+ group, and nine had not yet healed in the VFG− group (*n* = 19/28, 67.9%, 95% CI, 45.0 to 81.3%).

Regarding mid- and late-term revisions, by excluding occurrences in the first 2 years of follow-up, the revision rate was significantly lower in the VFG+ group (log-rank test, *p* = 0.034) (Fig. 3B). In this situation, we observed a significant difference, with 100% 5-year survival for the VFG+ group vs. 71.2% (95% CI, 55.0 to 92.1%) for the VFG− group and 15- and 20-year survival rates of 81.5% (95% CI, 77.3 to 100%) vs. 45.6% (95% CI, 25.1 to 82.6%), respectively.

In the VFG+ group, two revisions were necessary: a nail material replacement (Henderson type 3) and an allograft fracture on non-union (Henderson types 2 and 3). In the VFG− group, 10 revisions were necessary, 50% of them for non-union (Henderson type 2) (one of them was successfully healed by the addition of a VFG at 106 months), 30% for stabilisation device failure or modification (Henderson type 3), and 20% for chronic infection (Henderson type 4) (one of them requiring allograft removal at 36 months).

For the overall cohort survival rate at 1 year, the rate of allograft revision was 67.4% (95% CI, 55.1 to 82.4%) excluding oncological causes (Additional file 1: Fig. S1).

Early revisions before 1 year of follow-up occurred in 10 patients (35.7%) in the VFG− group, compared to 4 patients (22.2%) in the VFG+ group (Fischer test, *p* = 0.512). Before 2 years of follow-up, revisions occurred in 14 patients (50%) in the VFG− group, compared to 6 patients in the VFG+ group (33.3%) (Fischer test, *p* = 0.364).

**Fig. 4:**
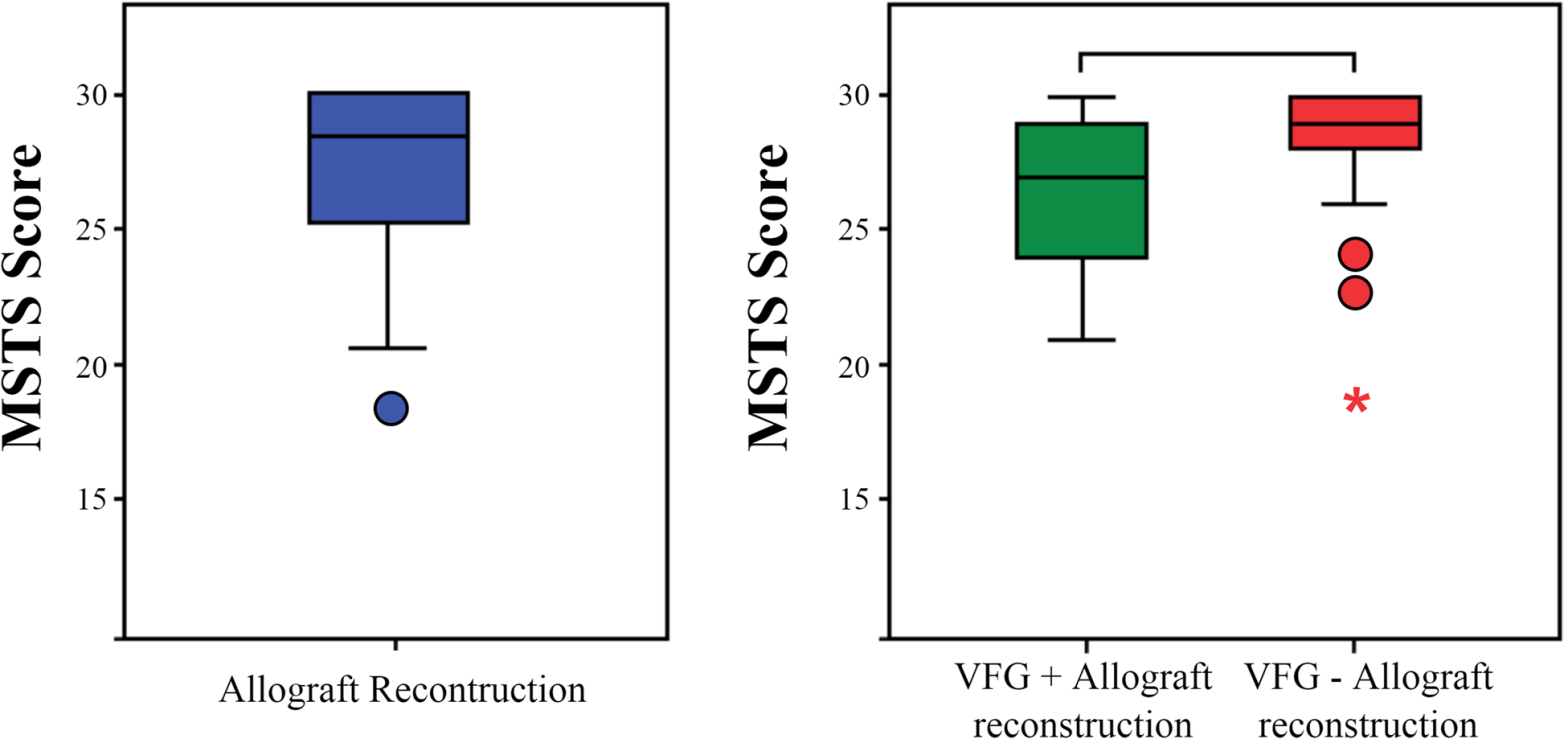
Box-and-whisker plots for the MSTS scores in allograft reconstruction and subgroup analysis for the VFG+ and VFG− groups; outliers are shown as a dot or asterisk, Student’s *t*-test

For the MSTS scores, we observed a mean MSTS score of 27.3 (18–30) in the cohort. The mean MSTS score was slightly lower in the VFG+ group than in the VFG− group (26.3 (21–30) vs. 28.0 (18–30); *p* = 0.060) (Fig. 4).

Cox model univariate analysis was performed for bone union, and it showed that VFG was a positive factor for the probability of bone healing, with a hazard ratio (HR) of 2.64 (95% CI, 1.28–5.44), *p* = 0.008. In the multivariate model analysis, VFG+ was again the most significant independent variable, with an HR of 6.53 (95% CI, 2.16–19.61), *p* = 0.001, while adjuvant radiotherapy was identified as a negative impact variable with an HR of 0.21 (95% CI, 0.06–0.74), *p* = 0.015 (Table 3).

**Table 3:** Univariate and multivariate (stepwise forward conditional method) Cox model regression for allograft bone union and revisions after 2 years of follow-up

	Bone union, HR^a^ (95.0% CI)	*p*-value	> 2 years revisions, HR^a^ (95.0% CI)	*p*-value
**Univariate analysis**				
Gender (male)	1.06 (0.56–1.99)	0.860	1.29 (0.38–4.32)	0.672
Age (years)^b^	0.99 (0.96–1.02)	0.460	1.01 (0.9–1.05)	0.790
Resection length (mm)^b^	1.00 (0.94–1.06)	0.967	0.98 (0.87–1.10)	0.705
Irradiated allograft	0.68 (0.35–1.32)	0.256	**3.57 (1.12–11.48)**	**0.031**
Adjuvant radiotherapy	0.32 (0.09–1.05)	0.061	2.63 (0.72–9.71)	0.144
Adjuvant chemotherapy	1.05 (0.51–2.14)	0.894	1.10 (0.33–4.55)	0.759
Diagnosis^c^				
CHS (^c^ref.)		0.841		0.661
OS	0.75 (0.28–1.98)	0.556	0.48 (0.14–1.67)	0.251
Ewing’s	0.84 (0.29–2.43)	0.742	1.20 (0.14–11.11)	0.866
Associated graft				
VFG−: *TG* (^c^ref.)		**0.026**		**0.025**
*CBG*	1.98 (0.85–4.49)	0.112	0.25 (0.03–2.00)	0.192
VFG+	**2.64 (1.28–5.44)**	**0.008**	**0.17 (0.04–0.80)**	**0.024**
Stabilisation				
Nail (^c^ref.)		0.149		0.095
Plate	1.17 (0.54–2.51)	0.690	0.79 (0.13–4.98)	0.814
Nail + plate	0.59 (0.27–1.30)	0.191	3.38 (0.83–12.82)	0.090
**Multivariate analysis**	Model *p*-value = 0.003		Model *p*-value = 0.010	
Adjuvant RT	**0.21 (0.06–0.74)**	**0.015**	**5.91 (1.19–29.38)**	**0.030**
Irradiated allograft			**6.00 (1.49–24.22)**	**0.012**
Associated graft: VFG−: *TG* (^c^ref.)		**0.004**		
*CBG*	1.89 (0.80–4.44)	0.145		
VFG+	**3.81 (1.68–8.64)**	**0.001**		

^a^Hazard ratio. ^b^Continuous variable. ^c^Reference variable in a categorical variable ≥ 3 levels

Regarding the revisions occurring after 2 years of follow-up, irradiated allografts were significantly more revised after 2 years in the univariate analysis, with an HR of 3.57 (95% CI, 1.12–11.48), *p* = 0.031. VFG+ was significantly associated with a lower revision risk and an associated HR of 0.17 (95% CI, 0.04–0.80), *p* = 0.024. However, in the multivariate analysis model, there were only two significant variables: adjuvant radiotherapy, with an HR of 5.91 (95% CI, 1.19–29.38), *p* = 0.030, and the most significant variable: irradiated allografts with an HR of 6.00 (95% CI, 1.49–24.22), *p* = 0.012 (Table 3).

## Discussion

Regarding bone union, we observed a significantly higher union rate by Kaplan-Meier survival analysis for VFG+ compared to VFG− allografts (log-rank, *p* = 0.017). The median bone union rate was 12.0 months for the VFG+ group and 20.0 months for the VFG− group. This observation is in accordance with the multivariate analysis model, in which the vascularised fibula graft was the most significant variable linked to the bone union, with an HR of 3.81, *p* = 0.001. These results appear to be in line with the data in the literature: in their study of 11 reconstructions by allograft and vascularised fibula, Ogura et al. found a bone union in 91% of cases, with a mean time to union of 9.9 months [31], and Frisoni et al. also reported that a vascularised autograft can be a protective factor against delayed union [32].

In intercalary reconstruction analysis efficiency analyses, bone union delay and occurrence were critical, allowing full weight-bearing and decreasing the risk of revision for Henderson type 2 complications. For this objective, an associated VFG appeared to have a positive effect on our population. Devitalised bone grafts, using various methods (irradiated, alcohol inactivated, autoclaved tumour bearing bone) reported similar bone union healing delay in the literature, ranging from 11 to 14 months [14–16].

After the first 2 years of follow-up, in the mid- and long-term, once most early complications have occurred [33, 34], revisions were significantly less frequent in the survival analysis in the VFG+ group (log-rank, *p* = 0.034). These results were not confirmed; however, in our multivariate Cox model analysis, in which the use of an irradiated allograft (HR=6.00, *p* = 0.012) was the most significant independent variable and appeared to have a more pronounced effect on mid- and late-term revision risk. It should be noted that almost half of the TG associated in the VFG− group were performed using an irradiated allograft, and this might thus be a confounding factor, impeding the TG association results. Negative results associated with irradiation preparation are in line with previous concerns that revealed higher failure rates for irradiated compared to non-irradiated allografts, especially with tendinous structures, such as Achilles tendon [35] or anterior cruciate ligament reconstructions [36]. For diaphyseal allograft indications, the review by Costain and Crawford observed a trend in favour of fresh-frozen allografts, with fewer structural failures, albeit without a clear or strong significance [37]. Therefore, our study confirms this tendency and objectifies significantly more revision rates and allograft resorptions with irradiated allografts in intercalary femoral reconstructions. Allograft long-term resorption issues, as mentioned by several authors, may be an explanation for this mid- and late-term revision difference [38], as we found with the greater resorption risk.

In light of our results, we recommend avoiding the use of irradiated allografts in this indication. It appears to be safer to use a fresh-frozen allograft, as an irradiated allograft may have impeded the long-term allograft structural reliability in our study, with more Henderson type 3 complications (allograft or material fractures), higher revision rates, and higher resorption rates.

**Fig. 5:**
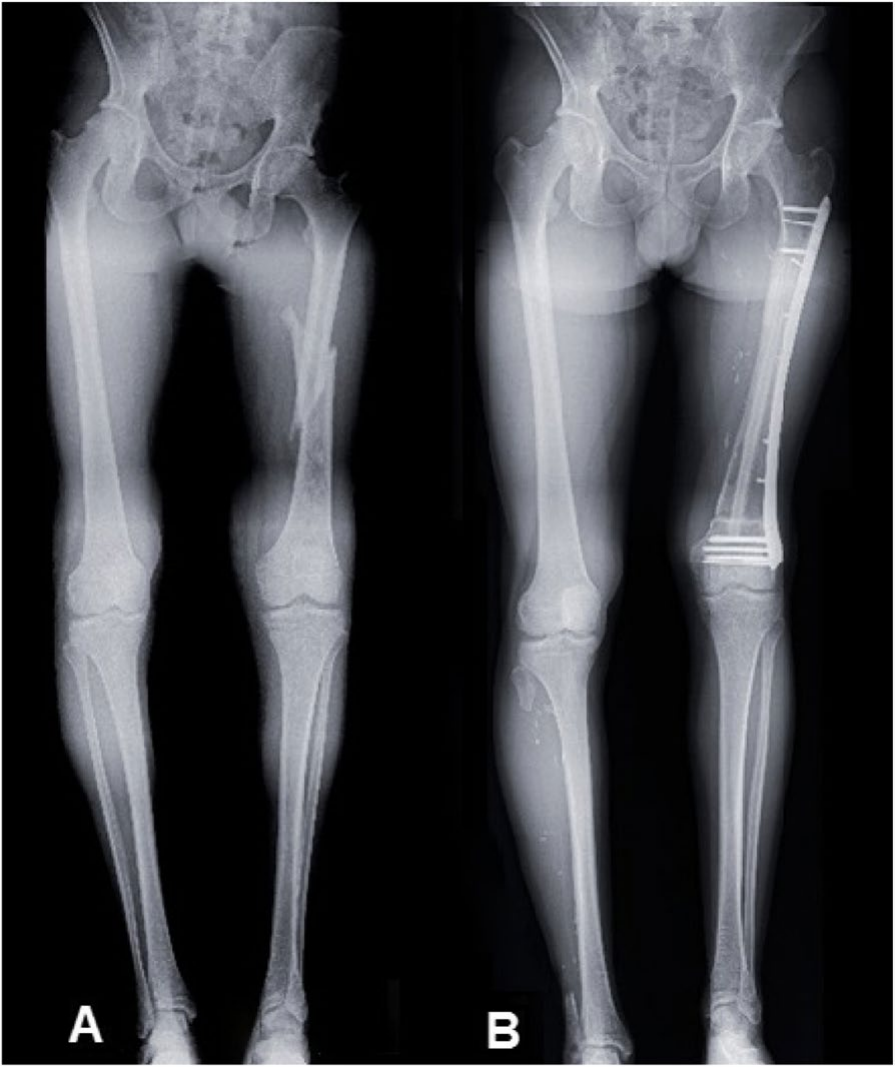
Intercalary femoral reconstruction for a high-grade fracture osteosarcoma using an allograft associated with a vascularised fibula graft and an LCP bridging plate. **A** Pre-operative pangonogram X-ray view. **B** Two-year follow-up X-ray view

Not surprisingly, adjuvant radiation therapy appeared to have a significant negative effect on bone union and mid- and late-term revision rates in the multivariate model (with HRs of 0.21, *p* = 0.015 and 5.91, *p* = 0.030, respectively). Its negative role has been well described in the literature, with higher septic complications and non-unions [39]. For Aponte-Tinao et al., radiation therapy is an allograft contraindication, and they hence perform intercalary endoprosthesis procedures in these cases [27]. Likewise, bridging plates can improve bone union, and they resulted in the greatest effect for less mid- and long-term revision in our univariate analysis, albeit without significance (Fig. 5). This result is in keeping with preclinical and human models, which consistently demonstrate the importance of rigid fixation in the allograft–host junction [40].

The functional results were satisfactory in our cohort, with an average MSTS score of 27.3 at the last follow-up (18–30). Likewise, Aponte-Tinao et al. observed a score of 27.0 at 68 months in a cohort of 83 femoral intercalary allografts [33]. The MSTS scores for VFG+ were slightly lower than for VFG−, but the difference was not significant: 26.3 (21–30) vs. 28.0 (18–30), respectively, *p* = 0.060. This trend may reflect the inherent iatrogenicity of fibular graft harvesting, as we observed two specific complications. Complications at the fibula harvest site are possible, and they are found in 6% of patients according to Bernd et al. and 20% according to Abed et al. [41, 42]. This may have led to the slightly lower functional score that we observed.

The main complications of this procedure reported in the literature are infection (0–14%), fractures (6–29%), and non-union (13–40%) [1, 43–45]. We did not find that there was a significant difference in the complications between the VFG+ group and the VFG− group. We observed a low rate of infections (Henderson type 4) (4.2%) in our entire cohort, while the literature reports rates of 5 to 18% in cases of allografts alone and 0 to 16% in cases of hybrid transplants [19, 46–48]. Since our study focused only on femoral reconstructions; this may explain why there were fewer infections than in studies that included tibial reconstructions, which are more prone to this type of complication. The Henderson type 2 and 3 complications were similar to those reported in the literature.

Despite the large number of cases, the homogeneous anatomical nature of femoral reconstructions and the long follow-up, this study has a number of limitations. Due to the multicentre analysis, this retrospective work can be limited to a certain degree by incomplete or imprecise documentation of postoperative events. Possible bias may mitigate our results, such as the young age of some of the patients, who could be subject to revisions for specific skeletal growth problems or who might have allograft bone union particularities [26]. As a retrospective study, there was no randomisation, and treatments (VFG+ or VFG−) were assigned depending on the surgeon’s preferences and skills. Moreover, statistical analysis might be interpreted with caution due to the small cohort. We chose to focus on mid- and late-term complications to increase the power of our analysis because most early complications may be the result of chemotherapy or radiation therapy, irrespective of the reconstruction choices. Finally, allografts with or without VFG result in comparable oncological outcomes, but they can differ in terms of the functional results as well as revisions and complications, which is why we excluded oncological revisions from our analysis, as suggested by Gundle et al. [49].

## Conclusion

Vascularised fibula grafts appear to have a positive effect on survival analysis and the multivariate Cox model, with significantly shorter times to bone union. In the survival analysis, mid- and long-term revisions after 2 years occurred significantly less in the VFG+ group, although the use of an irradiated allograft appeared to be a more detrimental confounding factor in our multivariate model, and caution should be taken with this allograft preparation with higher radiological resorption. At the last follow-up, the MSTS scores remained high in our cohort, with a mean score of 27.6. We did not observe a significant difference between our subgroups regarding this outcome. Based on our results, and despite possible fibula harvesting iatrogenicity and complexity, it is advisable to perform a vascularised fibula graft in a fresh-frozen allograft whenever possible. Future directions, such as personalised specific instruments, might be studied to facilitate and optimise intercalary allograft reconstruction using VFG.

## Supplementary Information

**Additional file 1: Figure S1.** Kaplan-Meier survival curve with failure defined as allograft revision excluding carcinologic causes. **Table S1**. Allograft resorption (ISOLS score) depending on the allograft type (fresh-frozen or irradiated), Fisher’s exact test: *p* = 0.006.

## Data Availability

The datasets generated and/or analysed for the current study are available from the corresponding author on reasonable request.
