# Supplementary material for: Intercalary allograft reconstruction following femoral tumour resection: mid- and long-term results and benefits of adding a vascularised fibula autograft
Source: World J Surg Oncol. 2022 Jun 13;20:195. doi: 10.1186/s12957-022-02650-x (PMC9195446; doi:10.1186/s12957-022-02650-x)
Supplement: Supplementary file 1 [file 12957_2022_2650_MOESM1_ESM.docx]

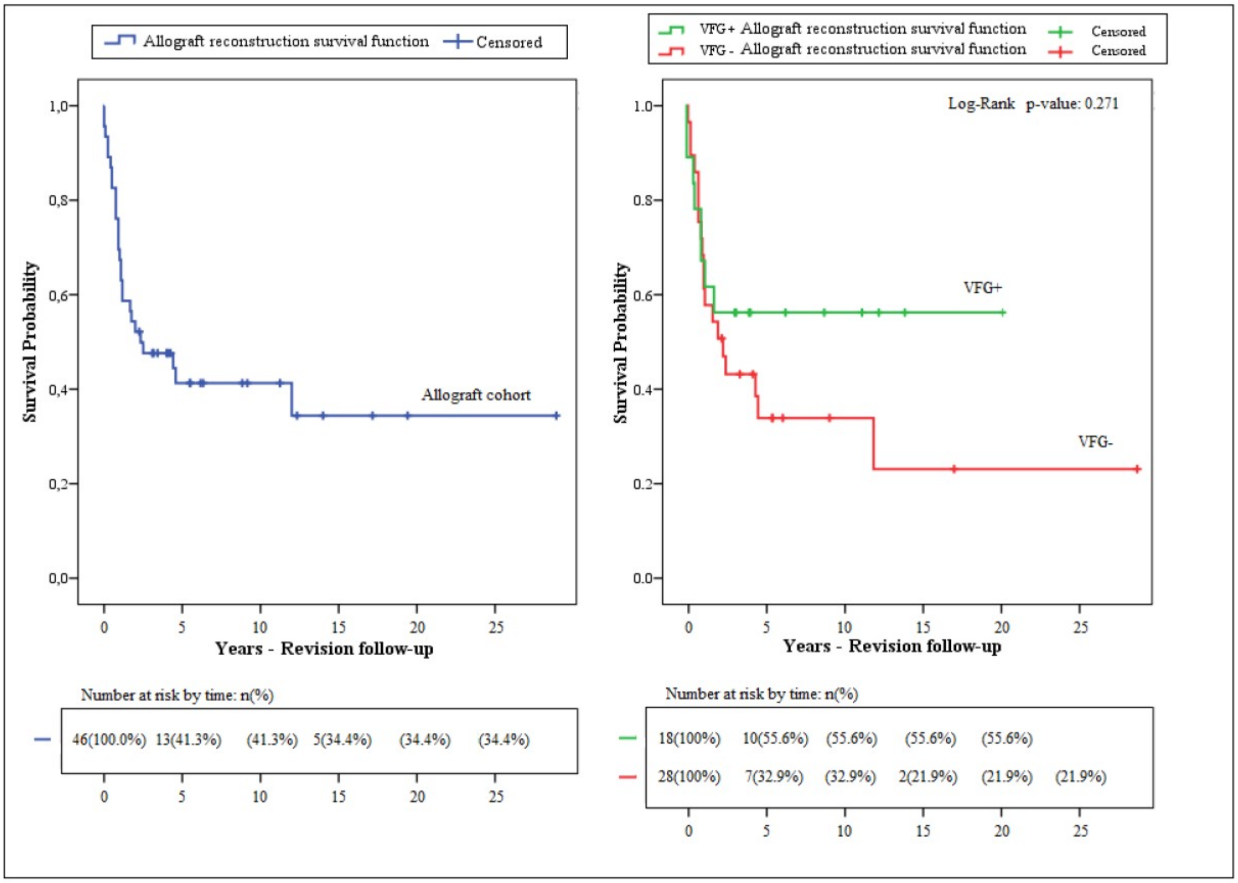

Additional Fig 1: Kaplan-Meier survival curve with failure defined as allograft revision excluding carcinologic causes.

	**Allograft resorption (ISOLS score)**				Total
	1/4	2/4	3/4	4/4	
Fresh-frozen allograft	26	4	2	0	32
Irradiated allograft	5	4	4	1	14
Total	31	8	6	1	46

Additional table 1: Allograft resorption (ISOLS score) depending on the allograft type (fresh-frozen or irradiated), Fisher's exact test: $p = 0.006$.
